# Assessing Preparedness for Self-Management of Oral Anticoagulation in Adults With the PERSONAE Scale: Protocol for a Development and Validation Study

**DOI:** 10.2196/51502

**Published:** 2025-02-26

**Authors:** Rosario Caruso, Gianluca Conte, Serenella Castelvecchio, Irene Baroni, Giulia Paglione, Giada De Angeli, Malgorzata Pasek, Arianna Magon

**Affiliations:** 1 Health Professions Research and Development Unit IRCCS Policlinico San Donato San Donato Milanese Italy; 2 Department of Biomedical Sciences for Health University of Milan Milan Italy; 3 Cardiac Surgery Department IRCCS Policlinico San Donato San Donato Milanese Italy; 4 Clinical Research Service IRCCS Policlinico San Donato San Donato Milanese Italy; 5 Department of Nursing, Faculty of Health University of Applied Sciences in Tarnów Tarnów Poland

**Keywords:** self-monitoring, self-management, oral anticoagulation, vitamin K antagonists, preparedness, validation

## Abstract

**Background:**

Optimal anticoagulation using vitamin K antagonists prevents strokes associated with atrial fibrillation and heart valve replacements. Preparedness for self-monitoring and self-management could improve outcomes, but this remains a challenge.

**Objective:**

This study aimed to outline the methodology for developing and validating the PERSONAE scale, a self-report measure designed to assess the preparedness for self-monitoring and self-management of oral anticoagulation in adult patients.

**Methods:**

This study comprises 2 main phases, and it adheres to the “COnsensus-based Standards for the selection of health Measurement INstruments” (COSMIN) guidelines for instrument development. The first phase involved the conceptualization of the PERSONAE scale, where a comprehensive literature review and a consensus meeting among experts were conducted to draft the initial items. Face and content validity were then established through an expert panel review. In the second phase (ongoing), a detailed sampling methodology will be used, targeting adult Italian patients on long-term oral anticoagulation. According to a performed simulation-based power analysis, the study aims to recruit a sample size of approximately 500 participants by using a combination of convenience and snowball sampling. Data collection will be facilitated through web-based surveys distributed through social media and patient networks, ensuring a wide and representative sample. Analytical procedures will include Mokken scaling analysis for item selection and confirmatory factor analysis to validate the scale’s structure. In addition, internal consistency will be assessed using Molenaar Sijtsma statistics.

**Results:**

The scale’s content derived from phase 1 (process completed in December 2023) is grounded in a comprehensive literature review and based on the assessments of a panel of 12 health care expert professionals. The PERSONAE scale derived from phase 1 encompasses 20 items reflecting essential behaviors needed to assess the preparedness for self-monitoring and self-management of oral anticoagulation. Each item obtained a content validity ratio higher than 0.67, which is the critical content validity ratio indicating the minimum level of agreement among the experts for an item to be considered essential beyond the level of chance at a significance level of .05 for a 1-tailed test. From January 2024 to May 2024, we conducted the initial round of data collection and use Mokken scaling analysis to select items. A second round of data collection for confirmatory factor analysis was scheduled from June 2024 to September 2024, which will validate the scale’s unidimensional structure. We expect to achieve robust psychometric properties, including high internal consistency and validated constructs.

**Conclusions:**

The PERSONAE scale will be a valuable tool to assess patients’ preparedness for self-monitoring and self-management of oral anticoagulation. The study’s insights into technology-assisted learning preferences will inform the design of future educational interventions to enhance preparedness in adult patients.

**Trial Registration:**

ClinicalTrials.gov NCT05973240; https://clinicaltrials.gov/study/NCT05973240

**International Registered Report Identifier (IRRID):**

PRR1-10.2196/51502

## Introduction

Vitamin K antagonists (VKAs), such as warfarin, are a group of medicines used to thin the blood, preventing the formation of harmful blood clots that can lead to stroke [1]. They function by inhibiting Vitamin K-dependent coagulation factors, which are essential for blood clotting. This class of medications is particularly vital for reducing the risk of stroke associated with atrial fibrillation and heart valve replacements, especially when direct oral anticoagulants are unsuitable for certain individuals, such as adults with multiple morbidities [1]. The use of oral anticoagulants globally is on the rise, reflecting an increasing need to manage conditions predisposing individuals to increased thromboembolic risks. Currently, about 2 out of every 100 Italians are on oral anticoagulants [2], including both direct and indirect agents [3]. This increasing prevalence underscores the necessity for regular monitoring of prothrombin time and appropriate dose adjustments, as inadequate monitoring and dose management could lead to serious adverse reactions and complications. Several studies have highlighted the risks associated with insufficient monitoring in the last 2 decades [4,5]. For instance, it was highlighted that patients on warfarin who did not have their prothrombin time regularly monitored were at a higher risk of major bleeding complications [4]. A meta-analysis published in 2020 confirmed that with the rising number of patients on oral anticoagulants, there is a proportional increase in adverse events related to inadequate monitoring [5].

People on long-term oral anticoagulation can use point-of-care testing equipment (POCT) to monitor their blood clotting time, typically measured by the international normalized ratio (INR) [6]. In managing their anticoagulant medication, these individuals have 2 primary options: self-management, where they adjust their medication dosage based on a predetermined dose-INR schedule, allowing them greater autonomy and immediate response to INR readings, or self-monitoring, where they measure their INR levels independently but consult health care providers to make dosage adjustments [7]. This distinction is crucial as self-management empowers patients to take full control of their therapy, potentially improving their adherence and outcomes, while self-monitoring still relies on professional guidance for dose adjustments [8,9].

Numerous studies and systematic reviews have demonstrated that these self-testing approaches are equally effective, if not superior, to routine physician-managed anticoagulation monitoring, providing evidence that patient-led monitoring can enhance therapy management and patient satisfaction [8,9]. For instance, a systematic review reported that patients who self-managed their oral anticoagulation therapy had significantly better control over their INR levels and fewer thromboembolic events than those who relied on professional monitoring [10]. In this context, a solid randomized controlled trial found that self-management was associated with higher patient satisfaction and better quality of life due to increased control over their health [11]. However, the choice between self-management, self-monitoring, and physician-managed care should be individualized based on patient characteristics. Recent researches highlight that health-related quality of life in anticoagulated patients varies significantly, indicating how some patients may benefit more from self-management while others may find self-monitoring or physician-managed care more suitable [9,12,13]. In addition, the quality of anticoagulation control, traditionally assessed by the time in the therapeutic range (%TTR), is enhanced by using recommended coagulometers like CoaguChek XS, which are globally endorsed, particularly in response to the COVID-19 pandemic [14,15].

While self-monitoring and self-management approaches have garnered strong evidence for their effectiveness and cost-effectiveness, a Cochrane review highlighted the need for further research into factors influencing individuals’ preparedness for self-testing [8]. These factors include both psychological preparedness and preparedness in terms of objective equipment. Psychological preparedness encompasses patients’ self-management knowledge, motivations for medical attention, health literacy, self-efficacy, and perceived and actual support systems [13,16]. Specifically, patients’ knowledge about self-management affects their ability to understand and manage their treatment, while motivations for seeking medical attention could influence their commitment to regular monitoring. Health literacy impacts their ability to comprehend medical instructions and make informed decisions, and self-efficacy relates to their confidence in performing self-testing tasks [17]. Finally, perceived and actual support systems are crucial in providing the necessary resources and emotional support for effective self-management. Preparedness in terms of objective equipment involves access to and familiarity with POCT devices and other necessary tools. Evaluating preparedness for self-management and self-monitoring is therefore crucial in tailoring health care delivery, enhancing efficiency, and adequately preparing patients for self-testing [18]. The interaction between psychological preparedness and preparedness regarding objective equipment is critical. Patients who are psychologically ready but lack the necessary equipment, or vice versa, may not effectively self-manage their condition. Therefore, both aspects must be addressed to ensure successful self-management and self-monitoring.

In Italy, for instance, adopting self-testing is complicated by organizational and professional barriers, such as the cost of POCT devices and concerns regarding the loss of direct control over treatment management [12]. Addressing this gap is essential, as enhancing patient preparedness through targeted interventions could substantially improve the effectiveness and patient outcomes of self-management programs, ultimately transforming anticoagulation therapy practices globally. The PERSONAE scale specifically addresses these gaps by assessing domains of preparedness and providing its comprehensive measure for self-management and self-monitoring. Thus far, no measures of the preparedness for self-monitoring and self-management exist in relation to this population. Therefore, the aim of this protocol is to outline the methodological steps for the development and validation of the PERSONAE scale, a self-report measure intended to assess the preparedness for self-monitoring and self-management of oral anticoagulation in adult patients. The study is currently focused on the Italian adult patient population, carefully considering the scalability and adaptability of the PERSONAE scale for diverse age groups and international settings. In addition, it aims to pinpoint the most favored technology-assisted learning options among patients undergoing anticoagulation therapy. The PERSONAE scale is expected to improve existing self-management protocols and practices by identifying areas where patients may need additional support or resources after a detailed assessment of patient preparedness. The findings from this pioneering research will lay the groundwork for innovative technology-assisted educational programs designed specifically to boost preparedness for self-monitoring and self-management of oral anticoagulation, marking a significant advancement in patient-centered health care solutions.

## Methods

### Study Design

This methodological, multiphase study comprises 2 main phases to develop and validate the PERSONAE scale for assessing the preparedness for self-monitoring and self-management of oral anticoagulation in adult patients. The protocol adheres to the “COnsensus-based Standards for the selection of health Measurement INstruments” (COSMIN) guidelines for instrument development [19].

Phase 1 focused on conceptualizing the PERSONAE scale through a developmental methodological approach involving 3 primary steps. First, a literature review was conducted to identify all relevant elements related to the preparedness for self-monitoring and self-management of oral anticoagulation (anticipated between May 2023 and July 2023). This review was recently published elsewhere [13]. Second, a consensus meeting among developers was held to examine the findings of the literature review and endorse its synthesis (anticipated time: September 2023). Finally, the situations representing patients’ preparedness will be operationalized, and an external panel of experts, including nurses, physicians, and pharmacists, will determine the face and content validity of the newly developed scale (anticipated time: October 2023). Phase 2 focused on the validation procedures. Initially, the PERSONAE scale, containing items with acceptable content validity, was used for cross-sectional data collection from January to May 2024. The Mokken scaling analysis (MSA) procedure will be used to assess how the items in the first version of the scale behave in response to varying levels of the theoretical latent trait (preparedness). MSA is preferred over exploratory factor analysis in this study as it allows selecting items with the highest scalability, leading to a brief, unidimensional measure computed in a single score. Once a stable unidimensional PERSONAE scale is developed, a second round of cross-sectional data collection was performed from June to September 2024 to cross-validate the plausible unidimensional structure of the scale.

### Participants and Procedures

In phase 1, a panel of 8-12 experts, comprising nurses, physicians, and pharmacists, evaluated the relevance and clarity of the proposed items derived from the literature review (content validity). The experts provided written consent to use their sociodemographic and professional characteristics for scientific reporting, as content validity is closely linked to the characteristics of the involved experts. This phase cannot be anonymous to ensure transparency, and variables such as sex, age, academic title, experience in studies testing content validity, and experience in oral anticoagulation were collected.

Once adequate content validity was established (December 2023), data collection commenced at the IRCCS (Istituto di Ricovero e Cura a Carattere Scientifico [Institute for Research and Healthcare]) Policlinico San Donato, using 2 cross-sectional data collection rounds using web surveys. A convenience sampling procedure was used, disseminating the web surveys through social media campaigns, newsletters, and flyers of associations involving anticoagulated patients, such as “Associazione Italiana Cardiopatici Congeniti” (AICCA). Participants for the second round were selected based on the same approach, with communication to prevent overlapping responses from participants who completed the survey.

Quality control measures have been implemented to ensure the integrity and quality of the data collection process: automatic data validation checks are included in the survey software to ensure that all responses are complete and adhere to the required format; unique identifiers were used for each participant to prevent duplicate responses in the dataset; and after data collection, statistical analyses were performed to identify any outliers or inconsistencies in the responses, which were then reviewed for accuracy.

### Sampling Procedure

The sampling procedure will use a cloud-data recording system and web surveys disseminated through social media campaigns, newsletters, and flyers. The local study coordinator and the principal investigator will manage the sampling procedure following good clinical practice principles. An expected response rate of approximately 60% is anticipated, which is considered adequate [20]. This expectation is based on several considerations, including using targeted engagement strategies such as precontacting potential participants and leveraging multiple dissemination channels. In fact, this rate aligns with the upper bound of the confidence interval from a recent meta-analysis, which reported an average response rate of 44.1% across 1071 studies in educational research [20]. Studies achieving similar response rates are those that have successfully implemented strategies to engage participants actively.

### Sample Size

The sample size for phase 1 will be 8-12 experts. The sample size required for phase 2 to perform the Mokken scaling analysis was determined considering the 20 items derived from phase 1 (content validity phase). A simulation-based power analysis was performed in R (R Foundation for Statistical Computing; library “Mokken”), as shown in Multimedia Appendix 1. The aim of the simulation was to estimate a sample size that would provide sufficient statistical power, set at 0.8, to detect a minimum H coefficient of 0.3, indicative of a moderate scale according to Mokken’s hierarchy. The H coefficient in Mokken scaling analysis measures the strength of the hierarchical relationship among items in a scale as it quantifies the extent to which items form a cumulative scale. A custom simulation function was created using R statistical software, with the following parameters defined: 20 items, initial sample (n= 200), number of simulations=100, desired power=80%, minimum H coefficient=0.3, and maximum number of iterations=30. For each simulation iteration, the function generated a matrix of binary data representing responses to the 20 items, with responses simulated under a simple random model (ie, the probability of success was 0.5 for each item). The results from the simulation suggested that a sample size of 500 patients would be needed to achieve the desired power of 0.8 for our MSA. The MSA procedure will help reduce the number of items to the first 10-15 items that define the preparedness for self-monitoring and self-management of oral anticoagulation in adult patients. The required sample size for the second round of data collection will be determined through Monte Carlo simulations, aiming to achieve a power of 0.8 for the confirmatory factor analysis (CFA) model.

### Eligibility Criteria

In phase 1, experts must have at least 1 year of experience in managing patients with VKAs, hold at least a master’s degree in nursing, pharmacy, or an MD degree, and have proficiency in the Italian language. Exclusion criteria for experts include conflicts of interest regarding the topic. In phase 2, patients aged 18 years and older will be included under oral anticoagulation treatment in an anticoagulation clinic for at least 3 months, with appropriate cognitive functioning and the ability to provide informed consent. Patients will be excluded based on more specific criteria to ensure the homogeneity and reliability of our data: those with a high Charlson Comorbidity Index (CCI>4), indicating severe concurrent medical conditions that could confound the study outcomes; patients undergoing any other experimental treatment affecting coagulation; and those with a history of noncompliance with medical regimens as reported by their health care providers.

### Study Procedures for Cross-Sectional Data Collections

Patients who agree to participate will access a web-based survey (SurveyMonkey) through social media channels, mobile messages, or emails. The invitation will include all relevant information about the study’s aim, methods, and tasks required for participation. Patients will confirm the inclusion and exclusion criteria during the initial access, and those who do not meet the criteria can opt out.

The data collection tools will include the PERSONAE scale derived from the content validity phase, a sociodemographic and clinical data form, and a graphic rating scale for assessing patients’ preferences for technology-assisted learning options. Sociodemographic data will include age, sex, marital status, educational level, and occupation, while clinical variables will encompass time in oral anticoagulation, clinical indication for anticoagulation, %TTR, and history of thromboembolic or bleeding complications in the last 3 months. These clinical variables are consistent with previous research, such as the studies that developed measures of knowledge in the same population [21,22], which examined similar sociodemographic and clinical factors to assess anticoagulation knowledge in the Italian population. This alignment ensures that our data collection is grounded in established theoretical frameworks and allows authors to generate hypotheses relevant to our target population.

### Data Analysis

The data analysis will involve the use of various statistical methods to develop and validate the PERSONAE scale.

For the first data collection round, MSA will be conducted using the “mokken” package in R [23]. MSA allows for the hierarchical evaluation of items, and the most relevant items that measure preparedness for self-monitoring and self-management of oral anticoagulation will be selected for the PERSONAE scale [24]. Loevinger’s coefficient of homogeneity function will be used to assess the scalability of the items. Monotonicity violations will be checked, and violations below 80 will be consistent with the requirements of a Mokken scale [23,25]. Loevinger’s coefficients of homogeneity (H) at the scale, item, and pairs of items level will be computed, where H values equal to or greater than 0.3, 0.4, or 0.5 indicate weak, moderate, or strong scales, respectively [23,25]. Item pairs violating monotonicity will be excluded, and floor and ceiling effects in data distribution will be assessed. Invariant item ordering (IIO) will be performed to further narrow down the pool of questions based on monotone homogeneity [25]. The IIO procedure will remove items one at a time until no significant violations are found. The Htrans (H^T^) coefficient will express the accuracy of the IIO selection, and H^T^>0.3 will denote proper IIO process and consistent Mokken scales. The final set of questions will be used to calculate ρ coefficients for reliability using the Molenaar-Sijtsma technique [26]. This approach is particularly adequate for assessing reliability in MSA because it provides a robust estimate of the internal consistency for nonparametric scales. The Molenaar-Sijtsma technique is well-suited for evaluating the scalability and reliability of the items within the scale, ensuring that the PERSONAE scale reliably measures the construct of preparedness for self-monitoring and self-management of oral anticoagulation.

CFA will be used to cross-validate the plausible unidimensional structure of the PERSONAE scale derived from the MSA, theoretically containing 10-15 items, using Mplus 8.1 (Muthén and Muthén, 2017). An unrestricted and unspecified model will be used with a maximum likelihood robust estimation of parameters. A confirmatory factor analysis with covariates will be used to estimate measurement invariance between subgroups if linear relationships between participant characteristics and the scale’s total score are detected [27]. Modification indices will be explored to identify items causing a violation of invariance. Items causing invariance will be evaluated for possible deletion from the final scale. The adequacy of confirmatory factor analyses in explaining sample statistics will be assessed using various fit indices, such as chi-square, chi-square/degree of freedom, comparative fit index, Tucker-Lewis index, and root mean square error of approximation [28]. Invariance testing based on different levels of CCI and other potential confounders will be considered if needed and is feasible to ensure that the scale functions equivalently across groups with varying levels of comorbidity and demographics.

Missing data will be managed using an available-case approach for missingness lower than 5% or regression imputations for missingness equal to or higher than 5%, assuming missing at random. The significance level for all analyses will be set at 5%. The software used for the analysis will include Mplus (version 8.1, Muthén and Muthén), IBM SPSS Statistics for Microsoft Windows version 27, and R (version 4.2.1, R Foundation for Statistical Computing).

### Ethical Considerations

The present protocol was approved by the Ethical Committee of Ospedale San Raffaele (protocol number 52/INT/2023). Ethical considerations will be carefully adhered to throughout the research process.

In the first stage of the study, which involves experts assessing the content validity [29,30], written informed consent will be obtained from each expert. They will be fully informed about the study’s objectives and their voluntary participation in the process. During the second part of the study, data collection will be carried out through anonymous web surveys. As per European Regulation 2016/679 and Legislative Decree 101/2018, this type of study ensures anonymity and does not require individual written informed consent from the participants [31]. However, to ensure transparency and participant understanding, a simple and clear presentation page will be provided to patients before they proceed to complete the web-based questionnaire. This page will explain the study’s purpose, the voluntary nature of their participation, and the anonymity of the survey.

The research team will prioritize the confidentiality and privacy of all participants involved in the study, adhering to the ethical principles outlined in the Declaration of Helsinki. Personal data will be handled with utmost care, and all data collected will be stored securely and accessible only to authorized personnel involved in the study. Any potentially identifying information will be removed from the dataset to ensure complete anonymity. Throughout the study, participants’ rights and welfare will be protected, and any potential risks will be minimized. The study findings will be reported and disseminated in a manner that preserves anonymity and confidentiality. Researchers will act responsibly, maintaining the highest ethical standards and ensuring that the study is conducted with integrity and respect for the participants’ rights and well-being.

## Results

The PERSONAE scale development and validation protocol has been fully funded by the “Fondazione Insieme per Vita agli Anni” with additional support from the Ricerca Corrente funding by the Italian Ministry of Health in 2023 (no specific grant number). The summary of the results is shown in Textbox 1.

**Results summarized per main categories.**

**Conceptual framework**

- Defined by the literature summarized in a literature review published elsewhere, describing self-care behaviors in patients on oral anticoagulant therapy [13].

**Focus of PERSONAE measure**

- Assess how ready patients feel about undertaking and managing the tasks associated with their anticoagulation therapy by translating the behavioral content identified in the review into terms of preparedness (refer to Multimedia Appendix 1).

**Item pool drafting**

- November 2023

**Expert panel for validation**

- Date of content validation: December 2023.
- Median age: 36 (IQR 29-45) years.
- Education qualifications: 25% (n=3) with a master of science, 25% (n=3) with a doctor of philosophy, and 50% (n=6) medical doctors.
- Specialties: 16.67% (n=2) in cardiology, 50% (n=6) in nursing, 16.67% (n=2) in pharmacy, and 16.67% (n=2) in internal medicine.
- Experience: All experts with more than 6 years in oral anticoagulation.
- Sex distribution: 66.67% (n=8) females.
- Conflict of interest: None reported (100%, n=12).
- Content: 50% (n=6) in nursing, 16.67% (n=2) in pharmacy, and 16.67% (n=2) in internal medicine.
- Experience: all experts with more than 6 years in oral anticoagulation.
- Sex distribution: 66.67% (n=8) females.
- Conflict of interest: none reported (100%, n=12).
- Content validity ratio (CVR): All 20 items obtained adequate CVR (refer to
  Multimedia Appendix 1
  for additional details).

**Scale question**

- “How prepared do you feel to independently monitor and manage your oral anticoagulation therapy for each of the following statements?”

**Rating scale**

- A 5-point Likert scale ranging from 1 (not at all prepared) to 5 (extremely prepared).

**Data collection for phase 2**

- Ongoing, anticipated completion by September 2024.

**Impact of PERSONAE scale**

- The PERSONAE scale will offer a comprehensive and validated tool to assess the preparedness of patients for self-monitoring and self-management of oral anticoagulation, aiding clinicians in tailoring interventions. Health care providers can empower patients to take an active role in their anticoagulation therapy, potentially improving treatment outcomes and reducing health care costs by encouraging safe and effective practices of self-monitoring and self-management.

**Dissemination of results**

- Upon successful validation, the results will be disseminated through peer-reviewed publications and presentations at international conferences. The research team plans to engage with professional societies and patient advocacy groups to promote the implementation of the PERSONAE scale in clinical practice globally.

The conceptual framework was defined by the literature summarized in a literature review published elsewhere, describing self-care behaviors in patients on oral anticoagulant therapy [13]. The focus on preparedness encapsulates the ability to perform specific self-care tasks and integrates the confidence and knowledge necessary to manage treatment proactively. In other terms, the version of the PERSONAE measure derived from phase 1 aims to assess how ready patients feel about undertaking and managing the tasks associated with their anticoagulation therapy by translating the behavioral content identified in the review into terms of preparedness (Multimedia Appendix 1). Once the pool of items was drafted (November 2023), an expert panel for validating the PERSONAE scale was involved, and it consisted of 12 professionals with diverse qualifications and backgrounds in health care. The process of content validation was performed in December 2023. The median age of the experts was 36 (IQR 29-45) years. Regarding higher education qualifications, 25% (3/12) held a master of science degree, another 25% (3/12) had earned a doctor of philosophy degree, and the remaining 50% (6/12) were medical doctors. Expertise among the panel members varied, with representation from multiple disciplines critical to managing oral anticoagulation therapy. Specifically, 16.67% (2/12) specialized in cardiology, the majority, 50.00% (6/12), were from nursing, 16.67% (2/12) practiced pharmacy, and 16.67% (2/12) were from internal medicine. When considering potential conflicts of interest, it was noted that 100% (12/12) of the experts reported none. All experts had substantial experience with oral anticoagulation, with each member having more than 6 years of experience in the field, which provided a solid foundation for their evaluative contributions. Regarding sex distribution, the panel was predominantly female, with 66.67% (8/12) of the members being women. All content validity ratio (CVR) of the 20 items obtained adequate content validity based on the evaluation of the experts (Multimedia Appendix 1; all observed CVR were equal or higher to the critical CVR, indicating the minimum level of agreement among the experts for an item to be considered essential beyond the level of chance at a significance level of .05 for a 1-tailed test).

The question guiding the self-report assessments derived from phase 1 is: “How prepared do you feel to independently monitor and manage your oral anticoagulation therapy for each of the following statements?” Therefore, the scale is designed for the patients. In the current form of the scale, patients are asked to rate their level of preparedness on a 5-point Likert scale ranging from 1 (not at all prepared) to 5 (extremely prepared). Therefore, the scale prompts patients to consider their proficiency across a spectrum of 20 activities (items) vital to the effective self-management of their treatment, from medication adherence to lifestyle adjustments and the use of technology.

The data collection for phase 2 is ongoing, and it is anticipated to be completed in September 2024. The PERSONAE scale will fill a critical gap by offering a comprehensive and validated tool to assess the preparedness of patients for self-monitoring and self-management of oral anticoagulation. Clinicians will be able to use this scale to identify patients who may benefit from self-testing approaches and tailor interventions accordingly. Health care providers can empower patients to take an active role in their anticoagulation therapy, potentially improving treatment outcomes and reducing health care costs by encouraging safe and effective practices of self-monitoring and self-management.

Upon successful validation, the results will be disseminated through peer-reviewed publications and presentations at international conferences. The research team plans to engage with professional societies and patient advocacy groups to promote the implementation of the PERSONAE scale in clinical practice globally.

## Discussion

### Principal Findings

This study outlines a methodological approach to developing and validating the PERSONAE scale, a self-report measure aimed at assessing the preparedness for self-monitoring and self-management of oral anticoagulation in adult patients. This novel scale aims to help health care providers discern patients’ preparedness for autonomous oral anticoagulation management, potentially leading to customized treatment strategies that enhance patient independence and treatment adherence. High scorers on the PERSONAE scale might be suitable for self-testing and adjusting their medication. At the same time, those with lower scores could benefit from targeted educational programs to improve their management capabilities, pending further research to establish specific scale cutoffs for optimal outcomes. It is important to note that the practical application of this scale and its cutoff points require empirical testing in future research to assess criterion validity and establish effective thresholds for different patient outcomes.

Phase 1 of the research protocol, based on a developmental methodological approach, was based on a literature review [13], followed by a consensus meeting among developers who have operationalized the literature into 20 measurable items of preparedness, leading to the initial version of the scale as per previous research focused on developing new self-report measures [32,33]. An expert panel consisting of 12 professionals from diverse health care disciplines (cardiology, nursing, pharmacy, and internal medicine) validated the content of the PERSONAE scale in December 2023. The experts, who brought a wealth of experience from their respective fields, ensured that the scale’s content was relevant and applicable to various professionals involved in anticoagulation management. This multidisciplinary approach enriched the scale’s content validity, ensuring comprehensive coverage of the necessary competencies for effective self-management. This phase was instrumental in creating a scale that is scientifically sound and clinically meaningful. This initial phase set a solid foundation for the subsequent validation phase, aiming to confirm that the scale accurately measures preparedness in a way that is predictive of patients’ ability to manage their anticoagulation therapy effectively.

In phase 2, validation procedures will be conducted to ensure the reliability and validity of the scale, and it is anticipated to end in September 2024. This process will require 2 cross-sectional data collections among Italian adult patients currently undergoing oral anticoagulation treatment. The collected data will be used to assess the scale’s psychometric properties and determine its performance in measuring preparedness for self-monitoring and self-management of oral anticoagulation [34]. More precisely, the first cross-sectional data collection will provide the needed power to perform the MSA procedure and determine item behavior in response to varying levels of the theoretical latent trait (preparedness) [35]. The second data collection will be based on the version of the scale updated from the analytics of the first data collection, and a CFA will be used to cross-validate the unidimensional structure of the PERSONAE scale. Cross-validation helps assess the generalizability of the scale’s results and allows researchers to determine if the validity holds true beyond the initial sample used for scale development [36].

### Limitations

Several limitations should be acknowledged. First, the sample size for the validation phase was based on a power-based simulation and Monte Carlo simulations, which may have inherent assumptions that could affect generalizability. Expanding future studies to include larger and more diverse populations could enhance the scale’s validity across varied demographics and age groups. Second, the use of web surveys for data collection may introduce response biases, particularly biases associated with self-reporting, such as recall bias and social desirability bias. Although measures such as ensuring participant anonymity and confidentiality are planned to mitigate these issues, the influence of these biases cannot be entirely eliminated. In addition, further exploration into potential sources of bias, including sampling biases due to nonrandom selection methods and the impact of the data collection medium on participant engagement and accuracy, is necessary. Limitations stemming from the analysis methods, such as potential confounding factors not accounted for in the statistical models, should also be critically evaluated. Future research should aim to address these limitations by adopting more robust sampling frameworks, using mixed methods for data collection, and applying comprehensive analytical techniques to provide a deeper understanding of the scale’s applicability and accuracy.

### Comparison With Previous Work

While previous studies have explored various components of oral anticoagulation therapy, such as patient knowledge, health literacy, and self-efficacy in self-management, the PERSONAE scale introduces a unique comprehensive measure [2,7-9,17]. It is the first self-report tool designed to assess both self-monitoring and self-management preparedness among adult patients on oral anticoagulation therapy. This distinction sets the PERSONAE scale apart from existing scales that typically focus on isolated aspects of anticoagulation management. Existing measures often independently evaluate patient knowledge or health literacy, which does not necessarily translate to practical, actionable patient readiness. In contrast, the PERSONAE scale encompasses a broader spectrum of preparedness, integrating aspects of knowledge, self-efficacy, and practical application capabilities into a single measure. This integrative approach not only aids health care providers in identifying well-prepared patients but also pinpoints those who might benefit most from targeted educational interventions. Furthermore, the development methodology of the PERSONAE scale enhances its robustness and applicability. The scale was crafted to ensure scientific soundness and clinical relevance through a rigorous, multiphase approach that included a literature review, expert consensus, and external validation. This methodological solidity supports its potential for widespread adoption and use in clinical settings, distinguishing it further from other tools that may lack such comprehensive validation.

### Conclusions

The PERSONAE scale represents a significant advancement in the management of oral anticoagulation therapy. It is the first comprehensive self-report measure specifically designed to assess the preparedness of adult patients for self-monitoring and self-management. This tool will enable health care providers to better identify patients ready to manage their treatment autonomously, possibly enhancing patient outcomes and optimizing therapy adherence. Our study aims to determine the scale’s robust psychometric properties, which underscore its reliability and applicability in clinical settings. The PERSONAE scale’s ability to encompass a broad spectrum of preparedness, from patient knowledge to self-efficacy and practical application skills, sets it apart from existing tools focusing on narrower anticoagulation management aspects. Looking forward, the PERSONAE scale has the potential to transform patient management in clinical practice globally. By facilitating the identification of patients suited for self-testing, the scale supports tailored educational interventions that can improve self-management skills among those less prepared. This approach promises to enhance patient autonomy and aims to reduce health care costs by improving treatment efficiency and satisfaction. Future research should focus on validating the scale across diverse patient populations to ensure its broader applicability. In addition, integrating the PERSONAE scale into routine clinical practice will be crucial for evaluating its impact on long-term patient outcomes and health care systems. Ultimately, the scale is poised to play a pivotal role in advancing patient-centered health care solutions in anticoagulation therapy.

## Appendix

Multimedia Appendix 1 Additional information.

## Abbreviations

%TTR: time in the therapeutic range
AICCA: Associazione Italiana Cardiopatici Congeniti (Italian Association of Congenital Heart Disease Patients)
CCI: Charlson comorbidity index
CFA: confirmatory factor analysis
COSMIN: COnsensus-based Standards for the selection of health Measurement INstruments
CVR: content validity ratio
IIO: invariant item ordering
INR: international normalized ratio
IRCCS: Istituto di Ricovero e Cura a Carattere Scientifico (Institute for Research and Healthcare)
MSA: Mokken scaling analysis
POCT: point-of-care testing
VKA: vitamin K antagonist
