# Supplementary material for: Assessing Preparedness for Self-Management of Oral Anticoagulation in Adults With the PERSONAE Scale: Protocol for a Development and Validation Study
Source: JMIR Res Protoc. 2025 Feb 26;14:e51502. doi: 10.2196/51502 (PMC11904369; doi:10.2196/51502)
Supplement: Multimedia Appendix 1 [file resprot_v14i1e51502_app1.docx]

**Supplementary File 1**. Draft of the scale (Phase One)

Note. The management of oral anticoagulation requires a comprehensive understanding and effective self-care behavior to prevent complications and ensure therapeutic efficacy. Recent advances in patient education and self-management strategies have underscored the importance of patient involvement in the monitoring and management of their treatment. The items included in this self-report measure are mainly derived from the scoping review by Magon, A., Hendriks, J. M., Conte, G., & Caruso, R. (2024)^[[1]](#footnote-1)^, which describes self-care behaviors in patients on oral anticoagulant therapy for non-valvular atrial fibrillation. The PERSONAE measure aims to assess how ready patients feel about undertaking and managing the responsibilities associated with their anticoagulation therapy by translating the behavioral content identified in the review into terms of preparedness. The focus on preparedness encapsulates the ability to perform specific self-care tasks and integrates the confidence and knowledge necessary to manage treatment proactively.

Question.

***How prepared do you feel to independently monitor and manage your oral anticoagulation therapy for each of the following statements?***

Instructions.

Please rate your level of preparedness to independently monitor and manage your oral anticoagulation therapy by selecting a number from 1 to 5 on the scale below where:

**1 = Not at all prepared: I feel completely unprepared and would not know where to start.**

**2 = Slightly prepared: I have a basic understanding but would need significant help to manage on my own.**

**3 = Moderately prepared: I feel somewhat confident but would be cautious about managing completely on my own.**

**4 = Very prepared: I am confident in my ability to manage most aspects of my therapy, though I might occasionally need assistance.**

**5 = Extremely prepared: I am fully capable and confident in my ability to manage all aspects of my therapy independently.**

Please consider all factors, including your knowledge of medication, ability to follow treatment plans, awareness of necessary lifestyle adjustments, and capability to recognize symptoms and take appropriate actions. Your response will help us understand your needs better and provide the right support to enhance your ability to manage your treatment effectively.

| The English version requires a full content validity process. The items presented here are solely reported for informative purposes because the content validity was performed only in the Italian version. | | | | | | |
| --- | --- | --- | --- | --- | --- | --- |
|  | **How prepared do you feel to independently monitor and manage your oral anticoagulation therapy for each of the following statements?** | 1  (Not at all Prepared) | 2 | 3 | 4 | 5  (Extremely prepared) |
| Item1 | Manage your medication schedule correctly, including taking the right doses at the right times. |  |  |  |  |  |
| Item2 | Maintain a diet that is compatible with your anticoagulation therapy, focusing especially on vitamin K intake. |  |  |  |  |  |
| Item3 | Recognize and respond to symptoms that may indicate problems with your therapy, such as unusual bruising or excessive bleeding. |  |  |  |  |  |
| Item4 | Make appropriate adjustments to your medication dosage based on INR values or professional advice. |  |  |  |  |  |
| Item5 | Implement necessary lifestyle changes to minimize risk activities that could affect your therapy, such as avoiding injuries. |  |  |  |  |  |
| Item6 | Use tools or devices to self-monitor your blood clotting time (INR) at home. |  |  |  |  |  |
| Item7 | Handle potential emergencies related to anticoagulation, such as bleeding events. |  |  |  |  |  |
| Item8 | Communicate effectively with healthcare providers about your management concerns and choices. |  |  |  |  |  |
| Item9 | Participate in decision-making about your treatment options and adjustments. |  |  |  |  |  |
| Item10 | Seek out and comprehend information about your condition and treatment alternatives. |  |  |  |  |  |
| Item11 | Attend scheduled follow-up appointments with healthcare providers |  |  |  |  |  |
| Item12 | Identify potential side effects of your oral anticoagulation medication. |  |  |  |  |  |
| Item13 | Use digital tools or apps to track your medication, diet, or INR levels |  |  |  |  |  |
| Item14 | Quickly contact the right healthcare professional or emergency services when an urgent issue arises. |  |  |  |  |  |
| Item15 | Educate and involve your family or caregivers in your anticoagulation management plan. |  |  |  |  |  |
| Item16 | Maintain consistency in activities that affect INR levels, such as diet and alcohol consumption. |  |  |  |  |  |
| Item17 | Handle uncertainties related to your health condition and treatment outcomes. |  |  |  |  |  |
| Item18 | Manage your anticoagulation therapy effectively, which includes monitoring your condition regularly and adjusting your treatment as needed. |  |  |  |  |  |
| Item19 | Understand medical information and instructions related to your anticoagulation therapy, including information provided by your doctor, nurse or pharmacist. |  |  |  |  |  |
| Item20 | Adapt to new technologies or changes in monitoring equipment that might be prescribed for managing your condition, such as using new INR monitors. |  |  |  |  |  |

| Italian version | | | | | | |
| --- | --- | --- | --- | --- | --- | --- |
|  | **Per ognuna delle seguenti situazioni, quanto ti senti preparato a monitorare e gestire in modo indipendente la tua terapia anticoagulante orale?** | 1  (Per nulla preparato) | 2 | 3 | 4 | 5 (Estremamente preparato) |
| Item1 | Gestire correttamente il tuo trattamento, inclusa l’assunzione dell’anticoagulante nelle giuste dosi e nei giusti momenti. |  |  |  |  |  |
| Item2 | Manterere una dieta compatibile con la tua terapia anticoagulante, concentrandoti in particolare sulla corretta assunzione di vitamina K con i pasti. |  |  |  |  |  |
| Item3 | Riconoscere e gestire i sintomi che potrebbero indicare problemi con la tua terapia, come lividi insoliti o sanguinamenti eccessivi. |  |  |  |  |  |
| Item4 | Fare gli aggiustamenti appropriati al dosaggio dei tuoi farmaci basandoti sui valori INR o sui consigli del personale sanitario. |  |  |  |  |  |
| Item5 | Implementare cambiamenti nello stile di vita necessari per minimizzare le attività a rischio che potrebbero influenzare la tua terapia, come evitare di ferirsi. |  |  |  |  |  |
| Item6 | Utilizzare dispositivi per auto-monitorare a casa il tuo tempo di coagulazione del sangue (valore di INR). |  |  |  |  |  |
| Item7 | Gestire le potenziali emergenze legate alla terapia, come eventi di sanguinamento. |  |  |  |  |  |
| Item8 | Comunicare preoccupazioni e scelte personali in modo efficace al personale sanitario |  |  |  |  |  |
| Item9 | Partecipare in modo attivo al processo decisionale che riguarda le opzioni di trattamento ed eventuali aggiustamenti di terapia se dovessero essere necessarie. |  |  |  |  |  |
| Item10 | Cercare e comprendere le informazioni sulle tue condizioni e sul trattamento. |  |  |  |  |  |
| Item11 | Partecipare agli appuntamenti di follow-up programmati con il personale sanitario |  |  |  |  |  |
| Item12 | Identificare i potenziali effetti collaterali del tuo farmaco anticoagulante orale. |  |  |  |  |  |
| Item13 | Usare strumenti digitali o app per tenere traccia della tua terapia, dieta o livelli di INR |  |  |  |  |  |
| Item14 | Contattare rapidamente il giusto professionista sanitario o i servizi di emergenza quando si verifica un problema urgente. |  |  |  |  |  |
| Item15 | Istruire e coinvolgere la tua famiglia o i tuoi caregiver nel piano di gestione della terapia anticoagulante. |  |  |  |  |  |
| Item16 | Mantenere una coerenza nelle attività che influenzano i livelli di INR, come la dieta e il consumo di alcol. |  |  |  |  |  |
| Item17 | Gestire le incertezze relative alla tua condizione di salute e agli esiti del trattamento. |  |  |  |  |  |
| Item18 | Gestire efficacemente la tua terapia anticoagulante, che include un monitoraggio regolare della tua condizione e l’eventuale aggiustamento del trattamento se necessario. |  |  |  |  |  |
| Item19 | Comprendere le informazioni sanitarie e le indicazioni relative alla gestione della tua terapia anticoagulante, incluse le informazioni ricevute dal tuo medico, infermiere o farmacista. |  |  |  |  |  |
| Item20 | Adattare alle nuove tecnologie o ai cambiamenti possibili gli strumenti d cui disponi per il monitoraggio dell’INR. |  |  |  |  |  |

| Content validity ratio (CVR) based on the assessment of an external panel of 12 experts | | | |
| --- | --- | --- | --- |
| item | Number of Rates of the items as “Essential” | CVR | Interpretation |
| Item1 | 10 | 0.67 | Adequate content validity |
| Item2 | 11 | 0.83 | Adequate content validity |
| Item3 | 12 | 1.00 | Adequate content validity |
| Item4 | 11 | 0.83 | Adequate content validity |
| Item5 | 11 | 0.83 | Adequate content validity |
| Item6 | 12 | 1.00 | Adequate content validity |
| Item7 | 10 | 0.67 | Adequate content validity |
| Item8 | 11 | 0.83 | Adequate content validity |
| Item9 | 12 | 1.00 | Adequate content validity |
| Item10 | 12 | 1.00 | Adequate content validity |
| Item11 | 11 | 0.83 | Adequate content validity |
| Item12 | 11 | 0.83 | Adequate content validity |
| Item13 | 12 | 1.00 | Adequate content validity |
| Item14 | 12 | 1.00 | Adequate content validity |
| Item15 | 11 | 0.83 | Adequate content validity |
| Item16 | 12 | 1.00 | Adequate content validity |
| Item17 | 11 | 0.83 | Adequate content validity |
| Item18 | 12 | 1.00 | Adequate content validity |
| Item19 | 10 | 0.67 | Adequate content validity |
| Item20 | 10 | 0.67 | Adequate content validity |
| Note: For a panel of 12 experts, based on Lawshe’s method, the critical CVR is 0.67. This value indicates the minimum level of agreement among the experts for an item to be considered essential beyond the level of chance at a significance level of 0.05 for a one-tailed test. | | | |

As shown below, the expert panel for the validation of the PERSONAE scale consisted of 12 professionals with diverse qualifications and backgrounds in health care. The median age of the experts was 36 years, with an interquartile range (IQR) of 29 to 45 years. Regarding higher education qualifications, 25% (n=3) held a Master of Science degree, another 25% (n=3) had earned a Doctor of Philosophy degree, and the remaining 50% (n=6) were Medical Doctors (MDs).

Expertise among the panel members varied, with representation from multiple disciplines critical to the management of oral anticoagulation therapy. Specifically, 16.67% (n=2) specialized in cardiology, the majority, 50.00% (n=6), were from nursing, 16.67% (n=2) practiced pharmacy, and 16.67% (n=2) were from internal medicine. When considering potential conflicts of interest, it was noted that 100% (n=12) of the experts reported none, ensuring an unbiased approach to the validation process. All experts had substantial experience with oral anticoagulation, with each member having more than 6 years of experience in the field, which provided a solid foundation for their evaluative contributions. Regarding gender distribution, the panel was predominantly female, with 66.67% (n=8) of the members being women.

| Characteristics of the involved experts (n=12) | | | |
| --- | --- | --- | --- |
| Age |  | N | % |
|  | Years (median, IQR) | 36 | (29-45) |
| Higher Education title | |  |  |
|  | MSc | 3 | 25.00 |
|  | PhD | 3 | 25.00 |
|  | MD | 6 | 50.00 |
| Expertise Area | |  |  |
|  | Cardiology | 2 | 16.67 |
|  | Nursing | 6 | 50.00 |
|  | Pharmacy | 2 | 16.67 |
|  | Internal Medicine | 2 | 16.67 |
| Conflict of interest | |  |  |
|  | None | 12 | 100.00 |
| Experience in oral anticoagulation | | |  |
|  | More than 6 years | 12 | 100.00 |
| Sex |  |  |  |
|  | Female | 8 | 66.67 |

**Sample size for Mokken Scale analysis**

**Code for the simulation in R:**

library(mokken)

# set of the parameters

n_items <- 20

n_samples <- 200

n_sim <- 100

desired_power <- 0.80

min_h <- 0.3

max_iterations <- 30

current_iteration <- 0

# Simulation

simulate_power <- function(n) {

data <- matrix(rbinom(n * n_items, 1, 0.5), ncol = n_items)

result <- coefH(data)

h_value <- result$h

return(ifelse(h_value > min_h, 1, 0))

}

powers <- replicate(n_sim, simulate_power(n_samples))

estimated_power <- mean(powers, na.rm = TRUE)

while ((is.na(estimated_power) || estimated_power < desired_power) && current_iteration < max_iterations) {

n_samples <- n_samples + 10

powers <- replicate(n_sim, simulate_power(n_samples))

estimated_power <- mean(powers, na.rm = TRUE)

current_iteration <- current_iteration + 1

cat(sprintf("Iteration %d: Sample size = %d, Estimated Power = %.2f\n", current_iteration, n_samples, estimated_power))

}

if (current_iteration == max_iterations) {

cat("Reached maximum iterations without achieving desired power.\n")

}

cat("Estimated sample size to achieve the desired power of 80%:", n_samples, "\n")

**Output:** Reached maximum iterations without achieving desired power.

> cat("Estimated sample size to achieve the desired power of 80%:", n_samples, "\n")

Estimated sample size to achieve the desired power of 80%: 500

1. Magon, A., Hendriks, J. M., Conte, G., & Caruso, R. (2024). Description of self-care behaviours in patients with non-valvular atrial fibrillation on oral anticoagulant therapy: A scoping review. European journal of cardiovascular nursing, zvae007. Advance online publication. https://doi.org/10.1093/eurjcn/zvae007 [↑](#footnote-ref-1)
